# Supplementary material for: Single-cell foundation modeling with species-native protein tokens links regenerative competence across frog and mouse
Source: bioRxiv. 2026 Jul 29:2026.07.27.740807. Preprint. [Version 1] doi: 10.64898/2026.07.27.740807 (PMC13483897; doi:10.64898/2026.07.27.740807)
Supplement: Supplementary file 1 [file NIHPP2026.07.27.740807v1-supplement-1.pdf]

bioRxiv preprint doi: https://doi.org/10.64898/2026.07.27.740807; this version posted July 29, 2026. The copyright holder for this preprint (which was not certified by peer review) is the author/funder. This article is a US Government work. It is not subject to copyright under 17 USC 105 and is also made available for use under a CC0 license.

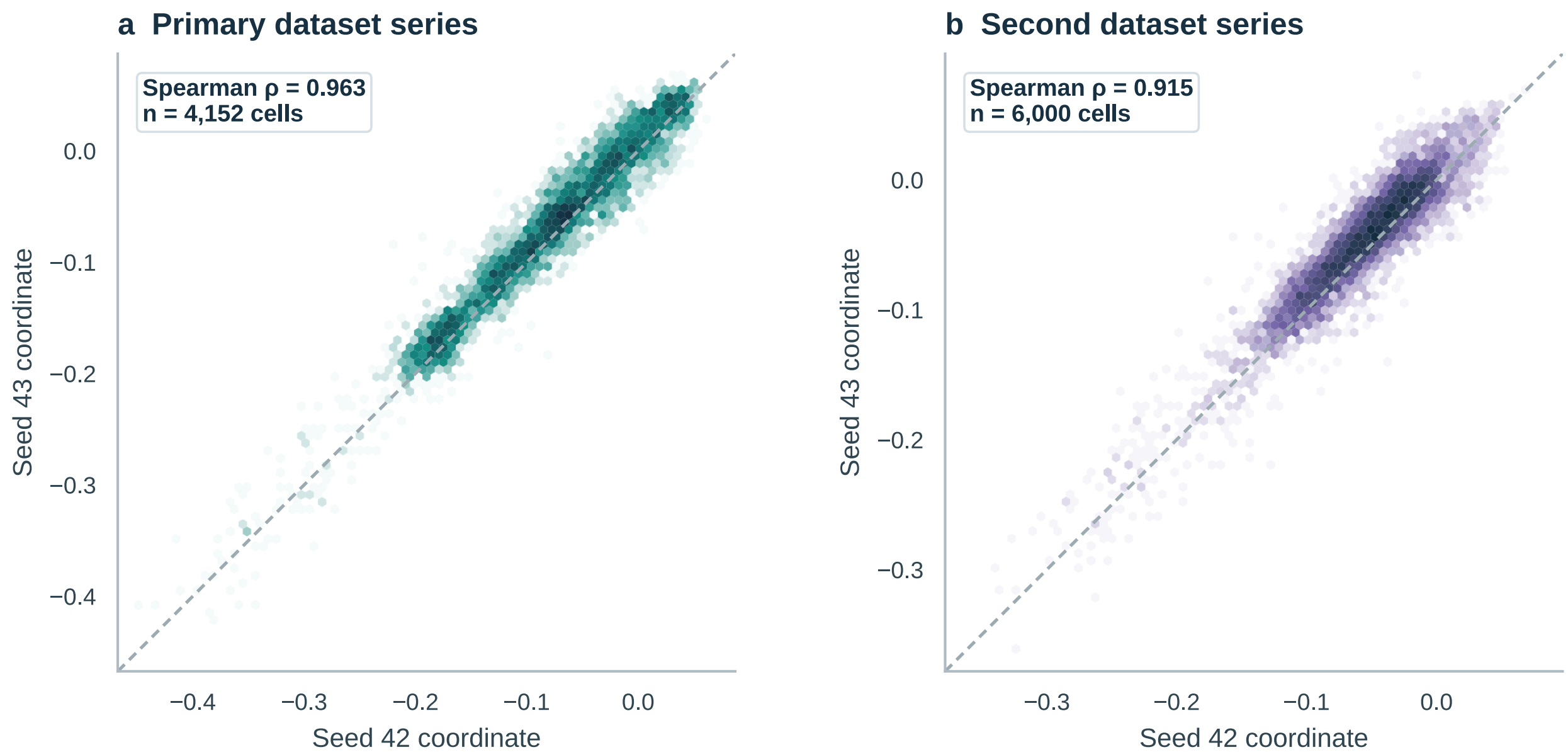

Supplementary Figure 1 | Cell-level token-sampling robustness in mouse. Each axis is the intermediate internal cosine coordinate—the dot product of a unit cell vector and its seed-matched unit ruler, bounded from −1 to +1—for seed 42 or 43 in the primary and second dataset series. These same-cell correlations assess computational reproducibility; biological conclusions compare deposited-library and condition-group summaries.

bioRxiv preprint doi: https://doi.org/10.64898/2026.07.27.740807; this version posted July 29, 2026. The copyright holder for this preprint (which was not certified by peer review) is the author/funder. This article is a US Government work. It is not subject to copyright under 17 USC 105 and is also made available for use under a CC0 license.

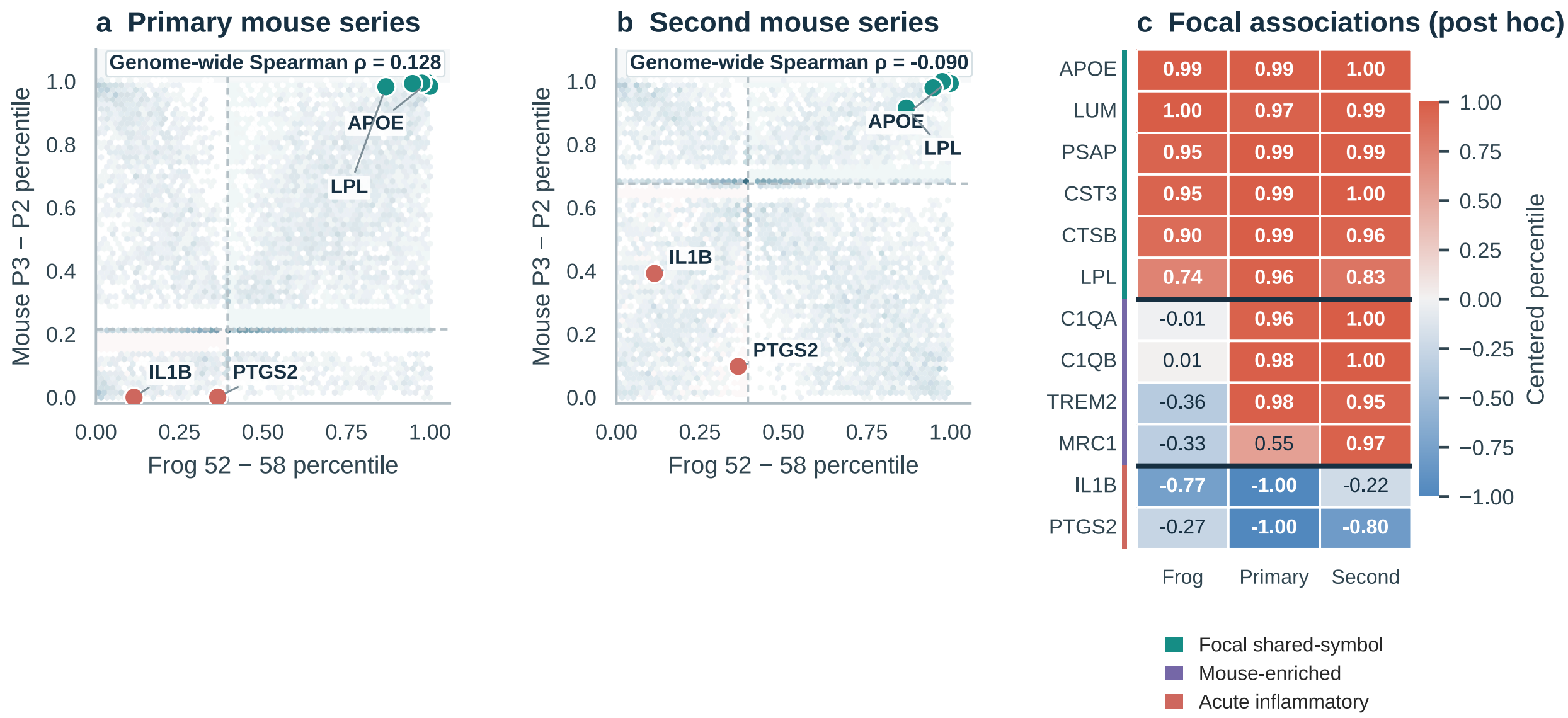

	Frog	Primary	Second
APOE	0.99	0.99	1.00
LUM	1.00	0.97	0.99
PSAP	0.95	0.99	0.99
CST3	0.95	0.99	1.00
CTSB	0.90	0.99	0.96
LPL	0.74	0.96	0.83
C1QA	-0.01	0.96	1.00
C1QB	0.01	0.98	1.00
TREM2	-0.36	0.98	0.95
MRC1	-0.33	0.55	0.97
IL1B	-0.77	-1.00	-0.22
PTGS2	-0.27	-1.00	-0.80

Supplementary Figure 2 | A focal frog-to-mouse tissue-remodelling bridge. a,b, Percentile-ranked shared-symbol expression effects for frog stage-52 versus stage-58 epidermis and immune-lineage P3 versus P2 effects in the primary and second mouse dataset series. Grey points show all 11,549 shared symbols and shaded quadrants show sign concordance; teal points mark selected non-translation genes positive in all three contrasts. **c,** Centered within-contrast percentiles distinguish focal lipid/lysosomal/extracellular-matrix-remodelling candidates from the mouse-dominant complement/*Trem2/Mrc1* component. The repair-module enrichment is reported in Supplementary Table 3. This orthology-restricted, post hoc analysis provides molecular interpretation of the transported ruler.

bioRxiv preprint doi: https://doi.org/10.64898/2026.07.27.740807; this version posted July 29, 2026. The copyright holder for this preprint (which was not certified by peer review) is the author/funder. This article is a US Government work. It is not subject to copyright under 17 USC 105 and is also made available for use under a CC0 license.

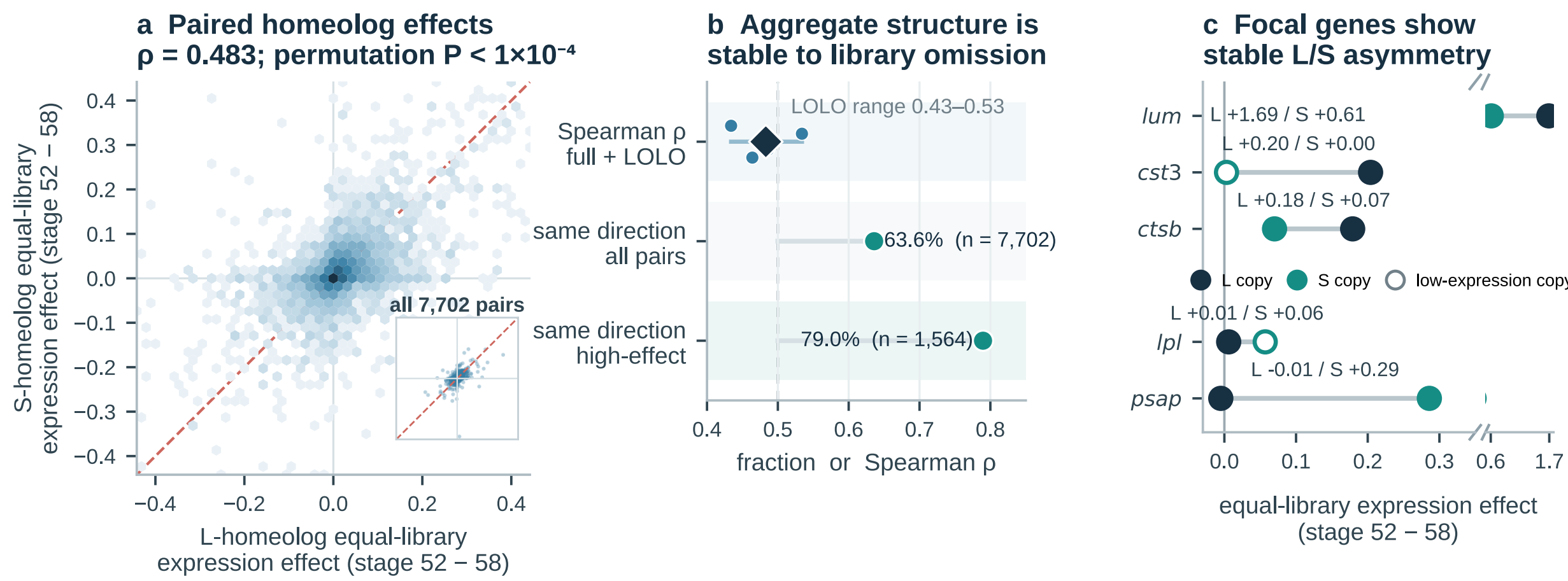

Supplementary Figure 3 | Homeolog-resolved expression structure in the frog limb competence contrast. a, Equal-library stage-52-minus-stage-58 log-normalized expression effects for 7,702 paired *Xenopus laevis* L/S homeologs; all pairs enter the statistics (Spearman ρ = 0.483; 10,000-permutation $P < 1 \times 10^{-4}$). **b,** Same-direction fractions across all and high-effect pairs, with full-data and leave-one-library-out correlations. **c,** L- and S-copy effects for focal genes whose asymmetry class was retained across all five library omissions; open symbols mark low-expression copies. This post hoc audit characterizes the homeolog structure retained by species-native UCE.

bioRxiv preprint doi: https://doi.org/10.64898/2026.07.27.740807; this version posted July 29, 2026. The copyright holder for this preprint (which was not certified by peer review) is the author/funder. This article is a US Government work. It is not subject to copyright under 17 USC 105 and is also made available for use under a CC0 license.

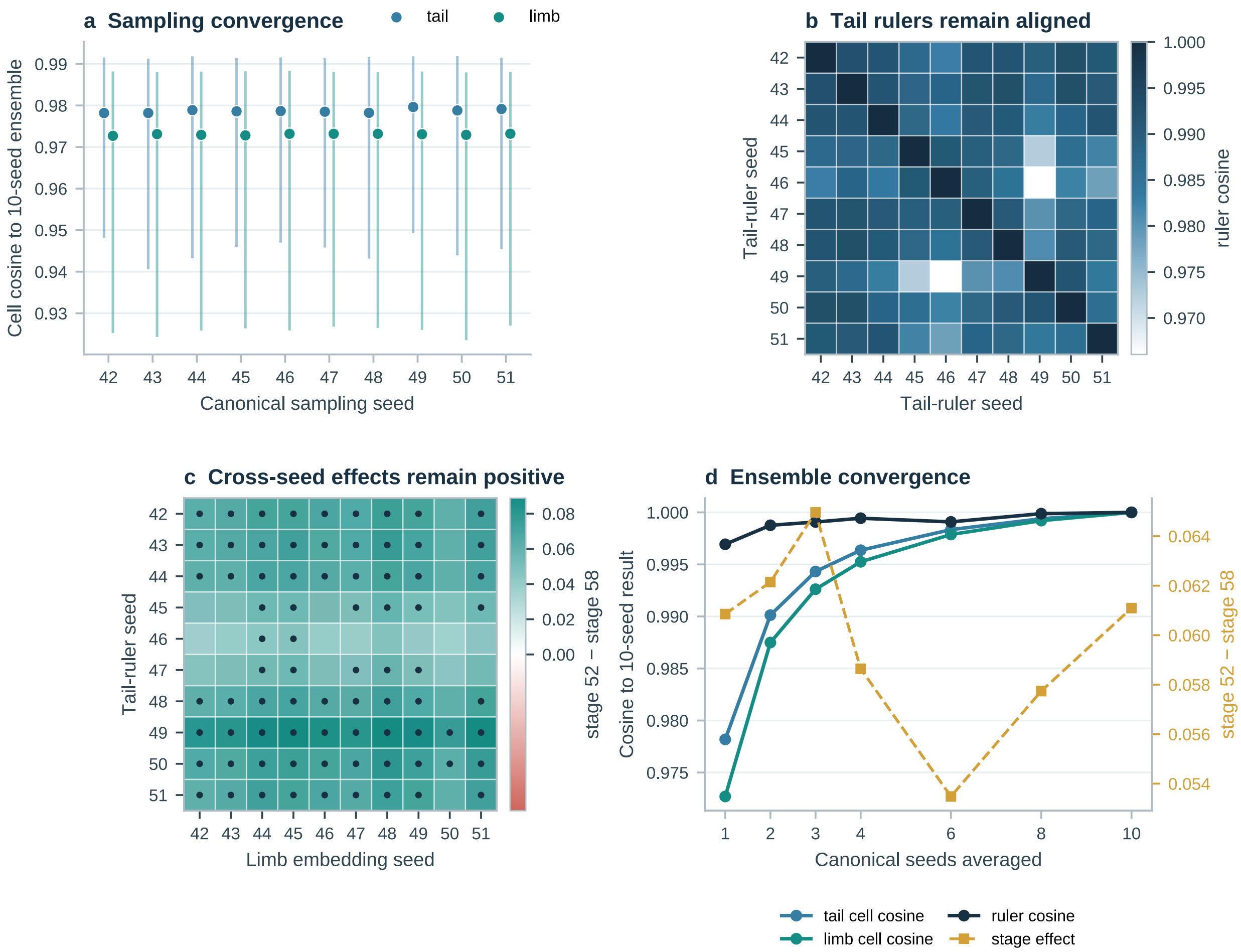

Supplementary Figure 4 | Ten-seed robustness of the frog tail-to-limb transfer. Canonical UCE token sampling was repeated for seeds 42–51. **a,** Same-cell cosine similarities compare each seed-specific UCE embedding vector with the unit-normalized ten-seed mean vector. **b,** Pairwise cosine similarities among the ten tail rulers. **c,** All 100 tail-seed-by-limb-seed combinations produced a positive raw stage-52-minus-stage-58 coordinate difference and complete early/late library separation; dots mark combinations with exact $P \leq 0.05$ and the expected median ordering. **d,** Convergence of same-cell embedding cosines, ruler cosine and the raw stage coordinate difference as seeds enter the ensemble. The ten-seed ensemble raw coordinate difference was 0.0611 with exact $P = 0.0339$. All ten leave-one-seed-out ensembles preserved direction, exact threshold and complete stage-52/58 separation. The mouse ruler remains based on the two prespecified seeds.

bioRxiv preprint doi: https://doi.org/10.64898/2026.07.27.740807; this version posted July 29, 2026. The copyright holder for this preprint (which was not certified by peer review) is the author/funder. This article is a US Government work. It is not subject to copyright under 17 USC 105 and is also made available for use under a CC0 license.

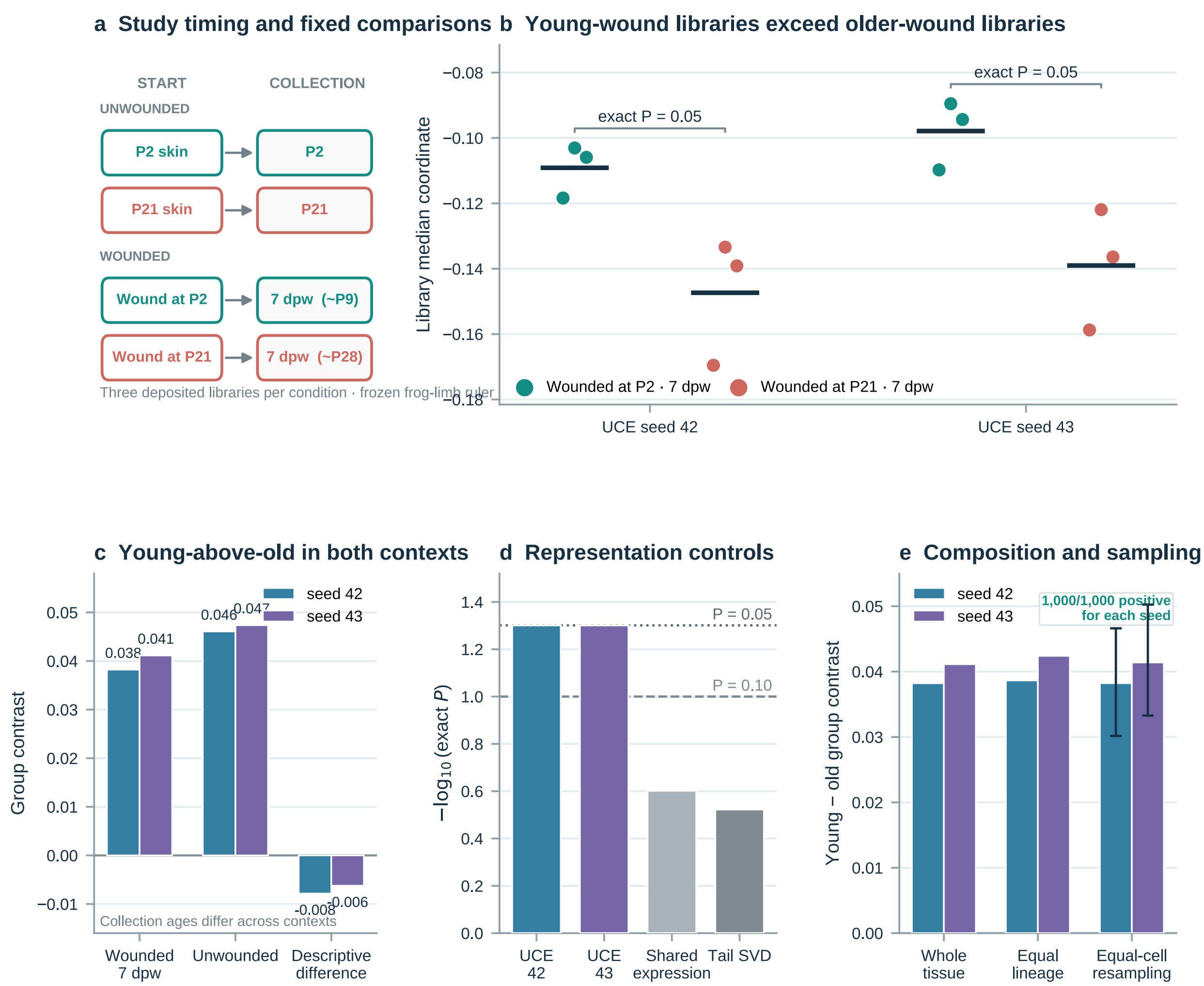

Supplementary Figure 5 | A frozen frog-limb ruler detects a developmental contrast across uninjured and wounded skin. a, GSE153596 timing. Unwounded skin was collected at PND2 or PND21; wounds initiated at PND2 or PND21 were collected seven days later (approximately PND9 or PND28), with three deposited libraries per group. **b,** Each point is a library median internal cosine coordinate; all libraries wounded at PND2 exceed all libraries wounded at PND21 for both UCE seeds, bars denote equal-library means and the exact one-sided probability is 0.05. **c,** Young-minus-old contrasts in wounded and unwounded contexts; their difference is descriptive because collection ages differ. **d,** Matched comparisons across 11,584 shared symbols place the UCE result against shared-gene expression and tail-trained SVD rulers. **e,** The wounded contrast remains positive after equal-lineage reweighting and in all 1,000 equal-cell resamples per seed. Raw contrasts are unitless coordinate differences, not probabilities.

bioRxiv preprint doi: https://doi.org/10.64898/2026.07.27.740807; this version posted July 29, 2026. The copyright holder for this preprint (which was not certified by peer review) is the author/funder. This article is a US Government work. It is not subject to copyright under 17 USC 105 and is also made available for use under a CC0 license.

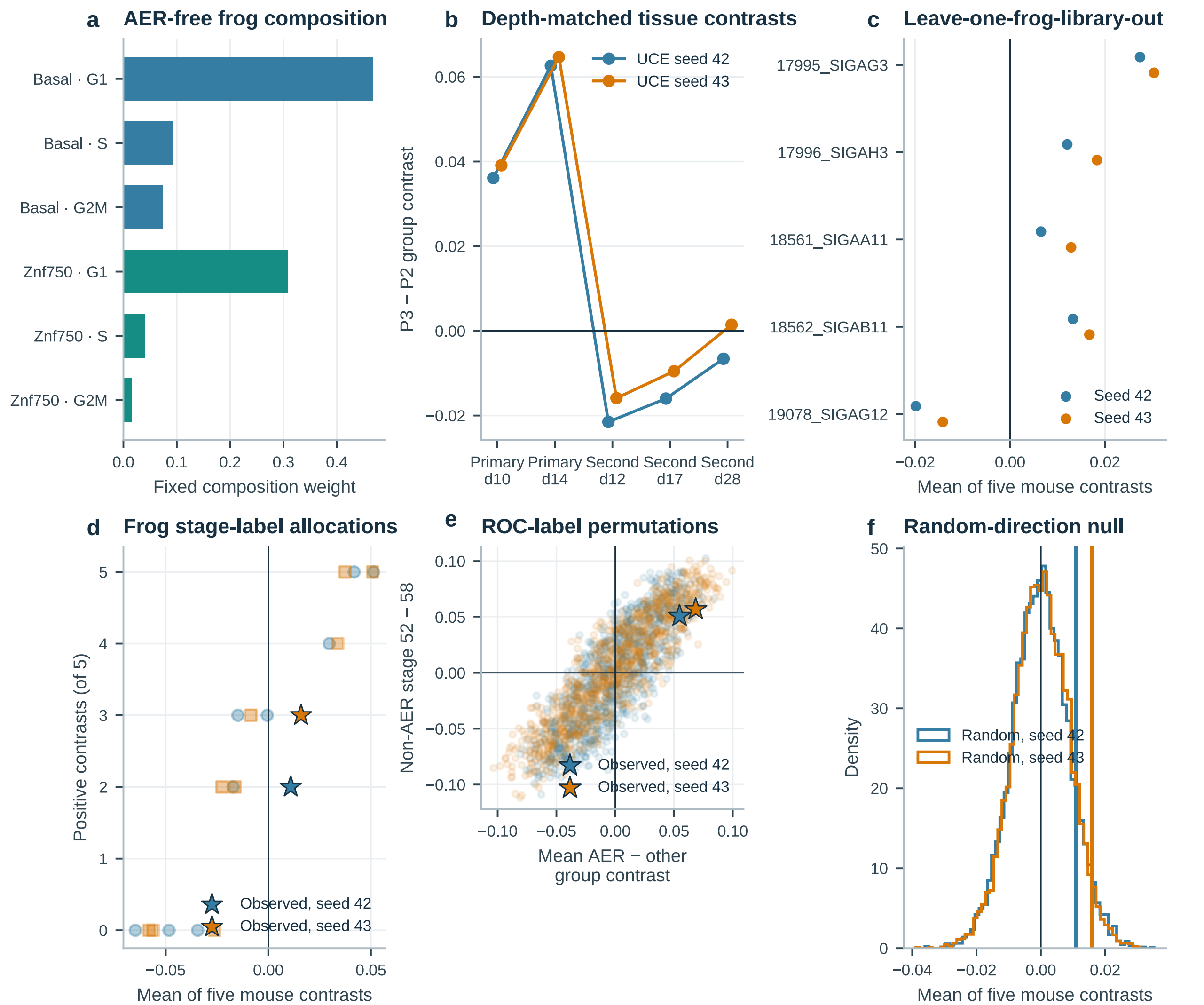

Supplementary Figure 6 | Controlled analyses resolve tissue-level transfer and upstream label evidence. a, Fixed weights across six AER-free frog epidermal strata. **b,** Pre-inference depth-matched tissue contrasts. **c,** Means of the five whole-tissue raw contrasts after leaving out each frog library. **d,** All ten frog-stage label allocations evaluated on the same five-contrast mean. **e,** Tail ROC labels permuted within each E-MTAB-7716 library; stars mark the observed AER separation and composition-standardized non-AER developmental contrast. **f,** The observed five-contrast whole-tissue mean against 10,000 random directions. Together, these controls motivate the progression from whole-tissue search to state-balanced macrophage analysis.

bioRxiv preprint doi: https://doi.org/10.64898/2026.07.27.740807; this version posted July 29, 2026. The copyright holder for this preprint (which was not certified by peer review) is the author/funder. This article is a US Government work. It is not subject to copyright under 17 USC 105 and is also made available for use under a CC0 license.

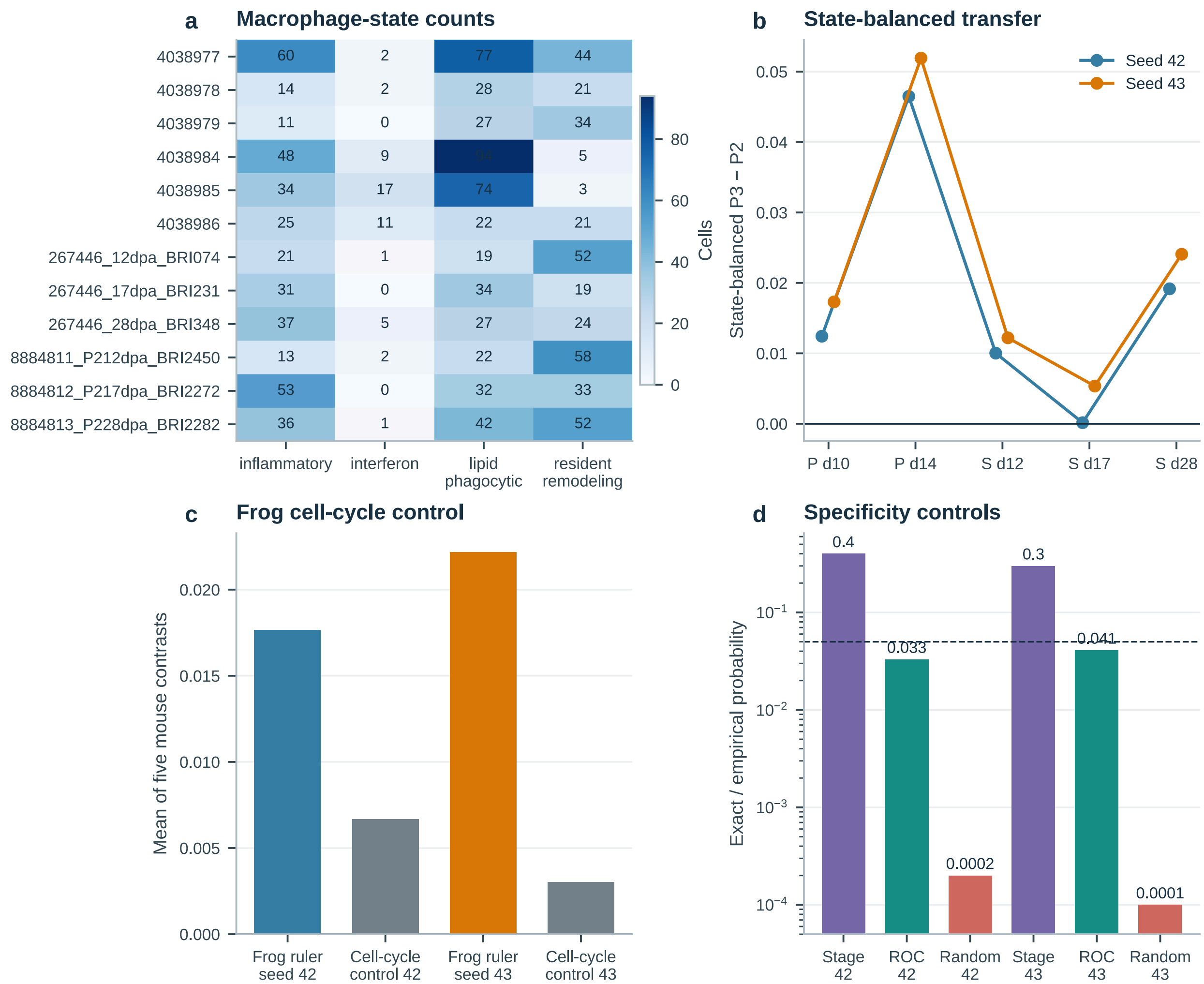

Supplementary Figure 7 | Fixed macrophage-state eligibility and summarized controls. a, Cell counts in four fixed macrophage states for every deposited mouse library; eligibility required at least ten cells in every library of a series. **b,** State-balanced P3-minus-P2 contrasts. **c,** Mean of the five state-balanced mouse contrasts for the frog competence ruler and frog cell-cycle control ruler. **d,** Exact frog-stage, joint ROC-permutation and random-direction probabilities on a logarithmic axis. The heat map displays every state count, including ineligible interferon and primary resident/remodelling strata.

bioRxiv preprint doi: https://doi.org/10.64898/2026.07.27.740807; this version posted July 29, 2026. The copyright holder for this preprint
(which was not certified by peer review) is the author/funder. This article is a US Government work. It is not subject to copyright under 17 USC 105 and is also made available for use under a CC0 license.

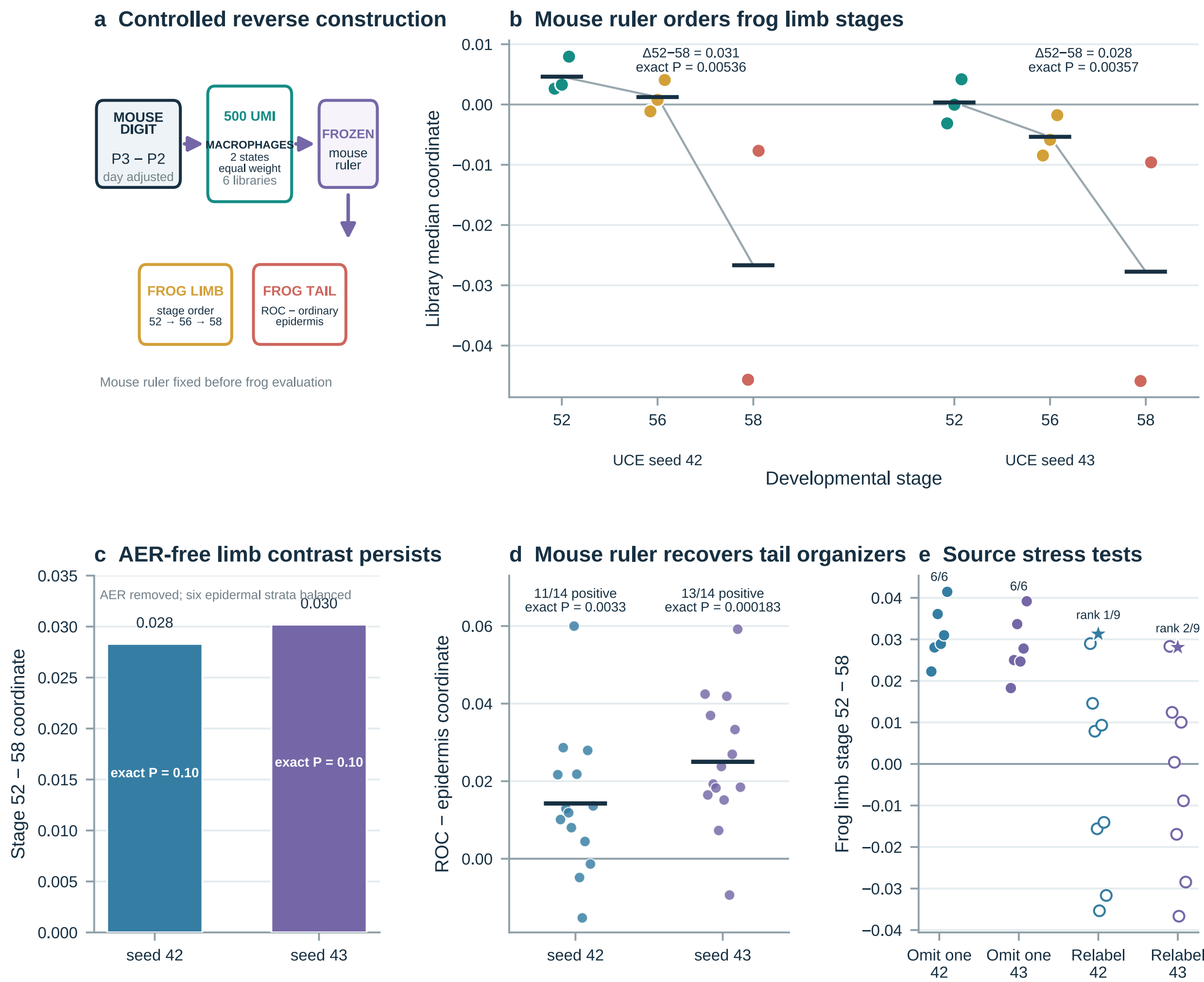

Supplementary Figure 8 | Reciprocal mouse-to-frog construction provides a geometric consistency check. a, The reverse ruler was built from six 500-UMI primary mouse digit libraries after giving lipid/phagocytic and inflammatory macrophage states equal weight and adjusting the P3-versus-P2 direction for collection day; frog data entered only after the ruler was frozen. **b,** Deposited-library median coordinates in the independent frog limb atlas. Both UCE runs produced the strict stage-52 > stage-56 > stage-58 order; horizontal bars denote equal-library stage means. **c,** Stage-52-minus-stage-58 differences after AER removal and six-stratum epidermal balancing. **d,** Within-library tail ROC-minus-ordinary-epidermis differences; horizontal bars denote equal-library means. **e,** Frog limb stage gaps after each mouse source-library omission (filled points) and across all nine within-day mouse outcome-label allocations (open points); stars mark the observed labels. The mouse-derived ruler was reconstructed independently for each UCE sampling run.

bioRxiv preprint doi: https://doi.org/10.64898/2026.07.27.740807; this version posted July 29, 2026. The copyright holder for this preprint (which was not certified by peer review) is the author/funder. This article is a US Government work. It is not subject to copyright under 17 USC 105 and is also made available for use under a CC0 license.

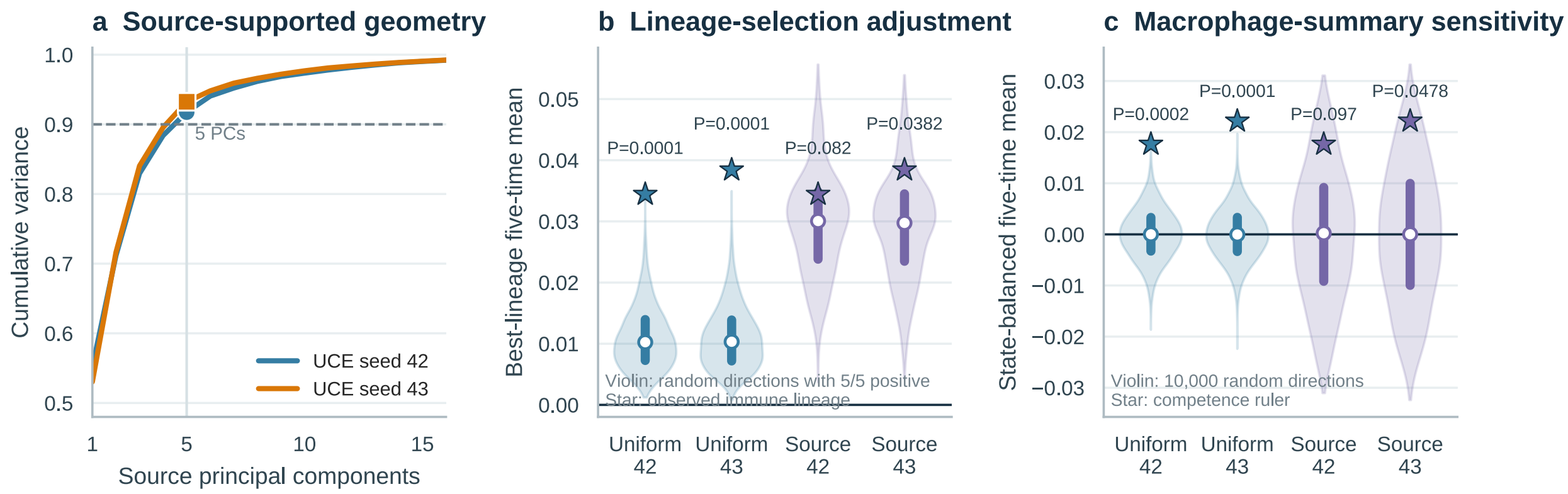

Supplementary Figure 9 | Selection-aware and source-supported controls calibrate ruler specificity. a, Cumulative variance of 30 AER-free frog stratum-by-library centroids; five principal components retain 91.7% and 93.2% of source variation in the two UCE runs. **b,** For every random direction, the complete lineage with the most positive P3-minus-P2 contrasts and then the largest five-time mean was selected. Stars mark the observed immune-lineage result; violins show uniformly sampled full-space directions or directions matched to frog source covariance that achieved five positive contrasts. **c,** The same direction families evaluated with the state-balanced macrophage summary fixed in advance. Each null contains 10,000 directions per UCE run, and displayed probabilities use the +1 correction.

bioRxiv preprint doi: https://doi.org/10.64898/2026.07.27.740807; this version posted July 29, 2026. The copyright holder for this preprint (which was not certified by peer review) is the author/funder. This article is a US Government work. It is not subject to copyright under 17 USC 105 and is also made available for use under a CC0 license.

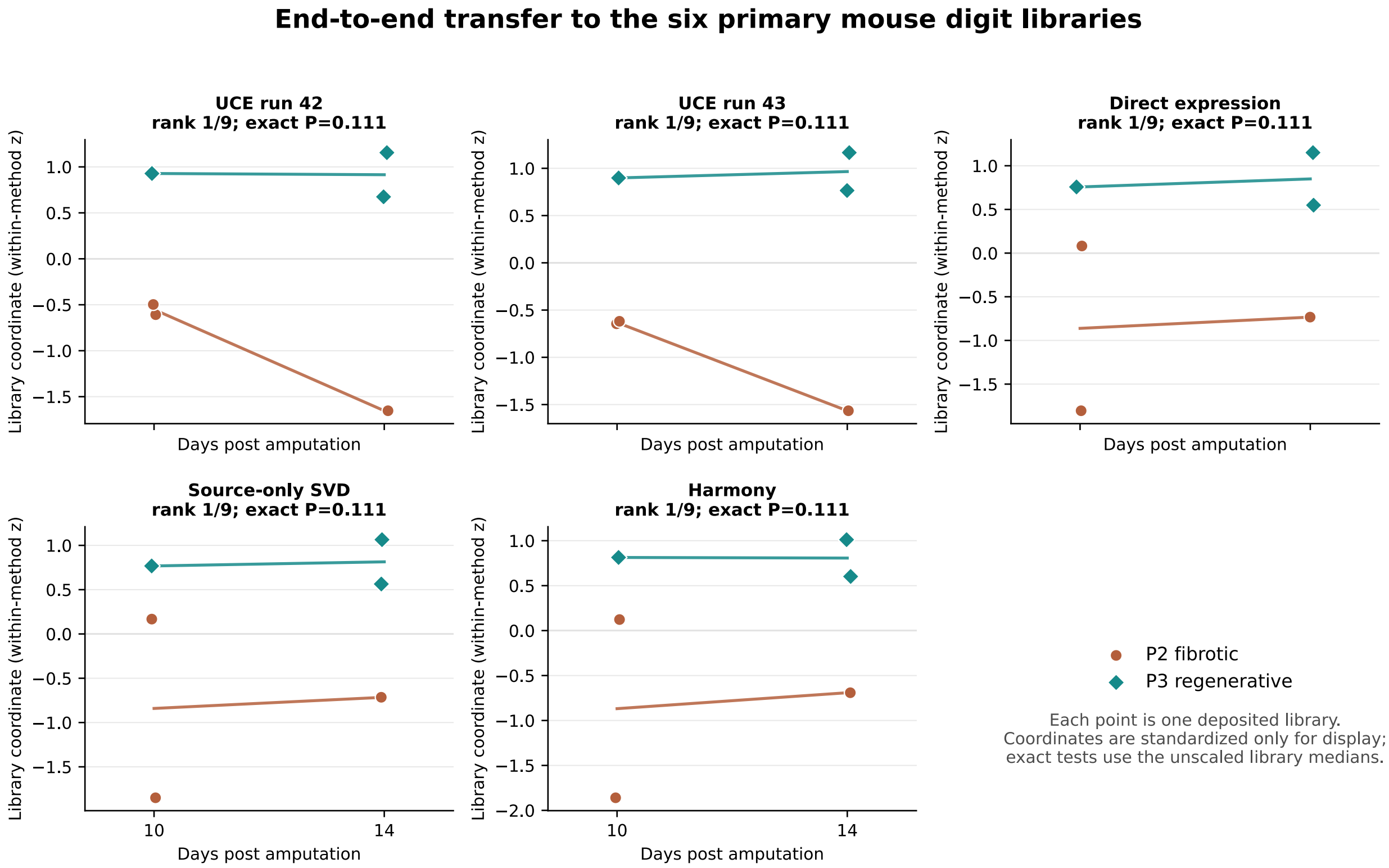

Supplementary Figure 10 | The primary mouse endpoint is reproduced by simpler representations. The complete frog-to-mouse chain was rerun independently for each representation. Every representation constructed its own frozen stage-52-minus-stage-58 ruler from the same five AER-free frog limb libraries, balanced across basal and Znf750 epidermal families and G1/S/G2M states, and applied it without outcome-label fitting to the same 4,152 mouse cells rarefied to 500 UMIs. Each point is one deposited library summarized by its median internal coordinate; coordinates are standardized within representation for display only, and the exact tests use unscaled library medians. All nine within-day outcome allocations were enumerated for each representation. Species-native UCE (both sampling runs), direct expression, source-only SVD and Harmony each place the observed labels first of nine, give complete 3-versus-3 separation and remain positive in all five source leave-one-out omissions. Harmony is target-aware but outcome-blind; direct expression and SVD are source-only. Raw coordinate magnitudes are not comparable across representations. Numerical values are given in Supplementary Table 4, and complete results and code are provided with the reproducibility materials.

bioRxiv preprint doi: https://doi.org/10.64898/2026.07.27.740807; this version posted July 29, 2026. The copyright holder for this preprint (which was not certified by peer review) is the author/funder. This article is a US Government work. It is not subject to copyright under 17 USC 105 and is also made available for use under a CC0 license.

Supplementary information

Supplementary Table 1 | Dataset and analysis inventory

Role	Accession	Species / tissue	Cells used here	Deposited libraries / donors	Biological contrast
Source-ruler construction	E-MTAB-7716	*X. laevis* tail epidermis	2,054	14	ROC versus ordinary epidermis
Independent limb-transfer test	E-MTAB-9104	*X. laevis* limb Tp63+ epidermis	9,142	18	AER identity; stage 52 versus 58 at 5 dpa
Expression-comparator context	GSE165816	*H. sapiens* diabetic-foot-ulcer epithelium	7,361	11 donors	9,960-symbol intersection and unlabelled Harmony context; outcomes unused
Mammalian primary series	GSE135985	*M. musculus* digit injury	4,152	6	regenerative P3 versus fibrotic P2 at 10 and 14 dpa
Mammalian second series	GSE293537 / GSE267446 / GSE143888	*M. musculus* digit injury	6,000	6	P3 versus P2 at 12, 17 and 28 dpa
Developmental skin / hair-follicle breadth test	GSE153596	*M. musculus* skin / hair follicle	8,400 sampled (66,921 after quality control)	12	young versus old skin; wounds initiated at PND2 or PND21 and collected 7 days later

Abbreviations. AER, apical ectodermal ridge; dpa, days post amputation; PND, postnatal day; ROC, regeneration-organizing cell.

bioRxiv preprint doi: https://doi.org/10.64898/2026.07.27.740807; this version posted July 29, 2026. The copyright holder for this preprint (which was not certified by peer review) is the author/funder. This article is a US Government work. It is not subject to copyright under 17 USC 105 and is also made available for use under a CC0 license.

Supplementary Table 2 | Principal results and controlled mouse analyses

Comparison or test	Seed 42	Seed 43	Inferential unit
Tail minimum leave-one-library-out direction cosine	0.9973	0.9975	held-out source library
Tail minimum eligible held-out AUROC	0.8728	0.8377	held-out source library
Limb AER-positive libraries	12 / 14	12 / 14	deposited library
Limb AER exact one-sided probability	0.000549	0.000244	library sign flip
Limb stage 52 versus 58 descriptive standardized effect	4.226	6.519	3 stage-52 versus 2 stage-58 libraries
Limb stage 52 minus 58 raw coordinate contrast	0.06085	0.06308	equal-library means; 3 stage-52 versus 2 stage-58 libraries
Limb exact stage-label probability	0.0393	0.0250	deposited 5-dpa library
Non-AER limb stage 52 minus 58 raw contrast	0.0391	0.0385	post hoc AER-exclusion sensitivity
Non-AER exact stage-label probability	0.0929	0.0786	deposited 5-dpa library
Initial mouse tissue screen: primary time-adjusted raw P3-minus-P2 contrast	0.0952	0.0956	deposited library, matched within time
Initial mouse tissue screen: primary exact within-time probability	0.1111	0.1111	6 deposited libraries; 9 allocations
Initial mouse tissue screen: series with positive mean raw contrast	2 / 2	2 / 2	series; second is accession confounded
Initial mouse tissue screen: positive matched contrasts	5 / 5	5 / 5	5 times nested within 2 dataset series
Depth-matched tissue audit: primary time-adjusted raw P3-minus-P2 contrast	0.0494	0.0519	AER-free source; 500-UMI pre-inference target
Depth-matched tissue audit: positive matched contrasts	2 / 5	3 / 5	scope boundary across 2 nested series

bioRxiv preprint doi: https://doi.org/10.64898/2026.07.27.740807; this version posted July 29, 2026. The copyright holder for this preprint (which was not certified by peer review) is the author/funder. This article is a US Government work. It is not subject to copyright under 17 USC 105 and is also made available for use under a CC0 license.

**Comparison or test**	**Seed 42**	**Seed 43**	**Inferential unit**
State-balanced macrophage mean of five raw contrasts	0.01765	0.02217	equal-state deposited-library summary
State-balanced macrophage positive matched contrasts	5 / 5	5 / 5	nested times; sign P = 1/32 corroborating
State-balanced macrophage primary exact probability	0.1111	0.1111	6 deposited libraries; 9 blocked allocations
Complete-lineage screen: immune mean and positive contrasts	0.03443 (5 / 5)	0.03842 (5 / 5)	outcome-informed selection among 3 complete lineages
Complete-lineage screen: endothelial / mural positive contrasts	2 / 5; 2 / 5	2 / 5; 2 / 5	same depth-matched screen
Uniform full-space null: selection-adjusted empirical probability	0.00010	0.00010	10,000 directions; lineage selection repeated per draw
Uniform full-space null: fixed macrophage empirical probability	0.00020	0.00010	10,000 directions; mean of five raw contrasts
Source-covariance null: selection-adjusted empirical probability	0.0820	0.0382	10,000 source-supported directions; lineage selection repeated
Source-covariance null: fixed macrophage empirical probability	0.0970	0.0478	10,000 source-supported directions; fixed state-balanced summary
Source covariance retained by 5 principal components	91.75%	93.24%	30 AER-free frog stratum-by-library centroids
Random directions with 5 / 5 positive contrasts	12.52%	12.75%	sign count alone is non-specific
Frog cell-cycle control: mean of five raw contrasts	0.00668 (5 / 5)	0.00301 (3 / 5)	AER-free cycling-minus-G1 ruler
State-balanced leave-one-frog-library-out rulers with positive five-contrast mean	5 / 5	5 / 5	6 / 10 retain all five positive contrasts

bioRxiv preprint doi: https://doi.org/10.64898/2026.07.27.740807; this version posted July 29, 2026. The copyright holder for this preprint (which was not certified by peer review) is the author/funder. This article is a US Government work. It is not subject to copyright under 17 USC 105 and is also made available for use under a CC0 license.

**Comparison or test**	**Seed 42**	**Seed 43**	**Inferential unit**
Exact source-stage allocations with mean ≥ observed	0.40	0.30	all 10 allocations of three early / two late libraries
Joint tail-ROC permutation bridge probability	0.0330	0.0410	1,000 within-library label shuffles
Reverse mouse ruler: frog limb stage 52 minus 58	0.03130; $P =$ 0.00536	0.02808; $P =$ 0.00357	8 deposited limb libraries; 560 stage allocations
Reverse mouse ruler: AER-free composition-standardized limb contrast	0.02830; $P =$ 0.10	0.03019; $P =$ 0.10	5 deposited limb libraries; 10 stage allocations
Reverse mouse ruler: tail ROC-positive libraries	11 / 14; $P =$ 0.00330	13 / 14; $P =$ 0.000183	within-library ROC-minus-epidermis sign flips
Reverse mouse ruler: positive frog limb gaps after source omission	6 / 6	6 / 6	one complete mouse library omitted per ruler
Reverse mouse outcome labels: observed frog limb rank	1 / 9	2 / 9	all within-day outcome allocations
Skin wounded-at-PND2 minus wounded-at-PND21 raw contrast	0.0382; $P =$ 0.05	0.0411; $P =$ 0.05	3 versus 3 libraries; all collected 7 days post wounding
Skin equal-lineage wounded-at-PND2 minus wounded-at-PND21 contrast	0.0386; $P =$ 0.05	0.0424; $P =$ 0.05	composition-reweighted deposited library
Skin equal-cell resamples positive	1,000 / 1,000	1,000 / 1,000	300 cells per library per resample
Skin unwounded PND2-minus-PND21 raw contrast	0.0461	0.0474	contextual; 3 versus 3 deposited libraries
Skin descriptive wounded-minus-unwounded developmental contrast	−0.00785	−0.00624	collection ages differ across contexts; not an isolated injury effect
Canonical ten-seed source–target combinations positive	100 / 100	100 / 100 separated	technical token-sampling audit
Ten-seed ensemble stage-52-minus-stage-58 raw contrast	0.0611; $P = 0.0339$		deposited 5-dpa library

bioRxiv preprint doi: https://doi.org/10.64898/2026.07.27.740807; this version posted July 29, 2026. The copyright holder for this preprint (which was not certified by peer review) is the author/funder. This article is a US Government work. It is not subject to copyright under 17 USC 105 and is also made available for use under a CC0 license.

Comparison or test	Seed 42	Seed 43	Inferential unit
Homeolog-pair expression-effect correlation	ρ = 0.483; 7,702 pairs		post hoc, equal-library expression audit

Scale note. Unless a row is explicitly labelled as a probability, AUROC, correlation or standardized effect, decimal ruler results are raw, unitless differences between library-level summaries of internal cosine coordinates. They are not probabilities, percentages or calibrated amounts of regenerative competence. Standardized effects are raw differences divided by pooled between-library standard deviations and can exceed 1 in magnitude.

Supplementary Table 3 | Targeted marker-set enrichment under an exact gene-label null

Previously defined marker set	Direction tested across frog and both mouse dataset series	Eligible / specified genes	Observed hits	Concordant background	Expected hits	Fold enrichment	Exact one-sided *P*	Bonferroni *P*
Repair / lipid / phagocyte	positive in all three	6 / 7	4	1,587 / 11,549	0.824	4.85	0.00423	0.00846

Supplementary Table 4 | End-to-end representation comparison at the primary mouse digit endpoint

Representation	Day 10 P3−P2	Day 14 P3−P2	Permutation rank	Exact *P*	Permutation *z*	Complete 3v3	Source LOLO positive
Species-native UCE, run 42	0.0361	0.0626	1/9	0.111	1.91	yes	5/5
Species-native UCE, run 43	0.0391	0.0647	1/9	0.111	1.93	yes	5/5

bioRxiv preprint doi: https://doi.org/10.64898/2026.07.27.740807; this version posted July 29, 2026. The copyright holder for this preprint (which was not certified by peer review) is the author/funder. This article is a US Government work. It is not subject to copyright under 17 USC 105 and is also made available for use under a CC0 license.

**Representation**	**Day 10 P3−P2**	**Day 14 P3−P2**	**Permutation rank**	**Exact *P***	**Permutation *z***	**Complete 3v3**	**Source LOLO positive**
Direct expression	0.0286	0.0280	1/9	0.111	1.59	yes	5/5
Source-only SVD	0.0711	0.0676	1/9	0.111	1.55	yes	5/5
Harmony	0.0538	0.0479	1/9	0.111	1.58	yes	5/5

Contrasts are time-adjusted P3-minus-P2 differences of deposited-library summaries in each representation's own units; magnitudes are not comparable across representations. The six-library design admits only nine within-day outcome allocations, so 1/9 = 0.111 is the finest attainable one-sided exact probability. Shared gene symbols for the expression-space comparators: 11,682. Source: 3 stage-52 and 2 stage-58 frog limb libraries. Target: 4,152 cells across 6 GSE135985 libraries. No target labels were used during representation fitting. Complete records are provided with the reproducibility materials.
